# Mutation E48K in PB1 Polymerase Subunit Improves Stability of a Candidate Live Attenuated Influenza B Virus Vaccine

**DOI:** 10.3390/vaccines9070800

**Published:** 2021-07-19

**Authors:** Jongsuk Mo, Stivalis Cardenas-Garcia, Jefferson J. S. Santos, Lucas M. Ferreri, C. Joaquín Cáceres, Ginger Geiger, Daniel R. Perez, Daniela S. Rajao

**Affiliations:** Department of Population Health, College of Veterinary Medicine, University of Georgia, Athens, GA 30602, USA; jm45001@uga.edu (J.M.); stivalis@uga.edu (S.C.-G.); jefferson.dasilvasantos@nih.gov (J.J.S.S.); lucas.matias.ferreri@emory.edu (L.M.F.); cjoaquincaceres@uga.edu (C.J.C.); imginger@uga.edu (G.G.); dperez1@uga.edu (D.R.P.)

**Keywords:** Influenza B, LAIV, PB1, vaccine stability

## Abstract

Influenza B virus (IBV) is a major respiratory pathogen of humans, particularly in the elderly and children, and vaccines are the most effective way to control it. In previous work, incorporation of two mutations (E580G, S660A) along with the addition of an HA epitope tag in the PB1 segment of B/Brisbane/60/2008 (B/Bris) resulted in an attenuated strain that was safe and effective as a live attenuated vaccine. A third attempted mutation (K391E) in PB1 was not always stable. Interestingly, viruses that maintained the K391E mutation were associated with the mutation E48K. To explore the contribution of the E48K mutation to stability of the K391E mutation, a vaccine candidate was generated by inserting both mutations, along with attenuating mutations E580G and S660A, in PB1 of B/Bris (B/Bris PB1att 4M). Serial passages of the B/Bris PB1att 4M vaccine candidate in eggs and MDCK indicated high stability. In silico structural analysis revealed a potential interaction between amino acids at positions 48 and 391. In mice, B/Bris PB1att 4M was safe and provided complete protection against homologous challenge. These results confirm the compensatory effect of mutation E48K to stabilize the K391E mutation, resulting in a safer, yet still protective, IBV LAIV vaccine.

## 1. Introduction

Influenza B virus (IBV) is an enveloped virus of the *Orthomyxoviridae* family with a negative-sense single-stranded RNA genome [1]. The IBV genome consists of eight segments, encoding at least 11 proteins [2,3]. IBV causes seasonal respiratory disease epidemics in humans. Although IBV can affect all age groups, studies demonstrate that individuals under the age of 18 are more susceptible to infection [4]. IBV infection can be severe in children and is associated with a high proportion of pediatric deaths related to influenza [5,6]. In most seasons, IBV infections are less common than influenza A virus (IAV) infections. However, the 2019–2020 season presented an unusually increased number of IBV infections, particularly early in the season, accounting for approximately half of the influenza infections in the U.S. (CDC Weekly U.S. Influenza Surveillance Report). IBV strains are classified into two antigenically distinct lineages, the B/Victoria (B/Vic) and the B/Yamagata (B/Yam) lineages [7,8]. The two lineages co-circulate globally, show minimal to no antibody cross-reactivity between them, and continue to evolve due to antigenic drift [7,8,9]. Thus, the development of effective vaccines against IBV is important to reduce disease burden, especially in vulnerable age groups.

Vaccines for influenza viruses mostly rely on antibody responses against the HA protein [10,11]. Most FDA-approved vaccines available against influenza are quadrivalent vaccines which incorporate strains from both IBV lineages, along with two subtypes of IAV (H3N2, H1N1). Trivalent vaccines that only contain one of the IBV lineages are also available [7,12,13,14]. In the United States, seasonal vaccines are available in the form of either recombinant proteins, inactivated influenza (IIV) vaccines, and/or live attenuated influenza virus (LAIV) vaccines. It is known that LAIVs can mimic a natural influenza infection, which can stimulate both mucosal and cellular immunity [15,16,17]. However, less than optimal vaccine efficacy of the FluMist LAIV in the 2013–2014 and 2015–2016 seasons, particularly in young children, prompted the CDC to temporarily recommend against its use in 2016 [18]. Although the CDC has since reinstated the recommendation for use of FluMist, this incident highlights the importance of developing other LAIV alternatives that could be more efficacious.

Previous work in our laboratory demonstrated that incorporation of three specific mutations (K391E, D581G, A661T) in addition to an HA epitope tag (HA tag) at the C-terminus of the PB1 protein segment and a single mutation (N265S) into the PB2 protein segment of IAVs results in a temperature sensitive (ts) phenotype in vitro, attenuates (att) IAVs in vivo, and provides effective protection against aggressive virus challenge in a variety of mammalian and avian animal models [19,20,21]. Following a similar approach, we previously generated a novel IBV att strain by introducing analogous mutations in the PB1 (K391E, E580G, and S660A with C-terminus HA tag) and PB2 (K267S) genes of the B/Brisbane/60/2008 (B/Bris) strain from the B/Vic lineage [22]. Although the PB2 K267S mutation could not be rescued and the PB1 K391E mutation was unstable, the remaining mutations in PB1 (E580G, S660A, HA tag) were stably maintained for at least 20 passages in Madin–Darby canine kidney (MDCK) cells or 15 passages in specific pathogen-free (SPF) eggs. Furthermore, these modifications resulted in attenuation of B/Bris in vitro and in vivo, and the resulting B/Bris PB1att strain protected mice against both homologous and heterologous challenge [22]. During stability tests, it was noted that the B/Bris PB1att that was serially passaged in MDCK cells retained the K391E mutation but showed an additional mutation in PB1, E48K. We hypothesized that E48K serves as a compensatory mutation to stabilize the K391E mutation while maintaining the attenuated phenotype.

To test this effect, we generated a new strain (B/Bris PB1att 4M) by incorporating E48K and K391E along with E580G and S660A mutations into the PB1 segment. The new B/Bris PB1att 4M strain, in which the HA tag was omitted, showed high stability in both MDCK cells and SPF eggs. In contrast, a strain containing the PB1 K391E (but not the E48K mutation, B/Bris PB1 3M) in the B/Bris PB1att background quickly acquired the E48K mutation after serial passage in eggs, further highlighting its importance as a compensatory mutation. Vaccination studies in mice showed that the B/Bris PB1att 4M strain was safe, immunogenic, and provided complete protection against homologous challenge.

## 2. Material and Methods

### 2.1. Tissue Culture Cells and Embryonated Chicken Eggs

Human embryonic kidney 293T cells, MDCK cells, and specific pathogen free (SPF) embryonated chicken eggs (Charles River, Norwich, CT, USA) were used for the generation of viruses through reverse genetics, virus amplification, and/or serial passages. Cells were cultured at 37 °C in a 5% CO_2_ environment in Dulbecco’s modified Eagle’s medium (DMEM) supplemented with 10% fetal bovine serum (FBS), 1% L-glutamine, and 1% antibiotic/antimycotic solution (Sigma-Aldrich St. Louis, MO, USA).

### 2.2. Cloning and Site-Directed Mutagenesis of PB1 Segment

The reverse genetics plasmid encoding the PB1 segment of the B/Bris att strain containing three amino acid mutations (K391E, E580G, and S660A) was previously described in [22]. The E48K mutation was introduced into the PB1 gene segment by site-directed mutagenesis with the QuikChange II XL kit (Agilent, Santa Clara, CA, USA) to generate the PB1 4M plasmid. Plasmids were grown in One Shot TOP10 *E. coli* cells (Invitrogen, Carlsbad, CA, USA) in the presence of ampicillin and stocks were prepared via the HiSpeed Plasmid Maxi Kit (Qiagen, Germantown, MD, USA). Plasmids were sequenced by Sanger sequencing with a specific set of primers (see below) to corroborate the mutations in the PB1 4M plasmid and to rule out spurious mutations.

### 2.3. B/Bris PB1att 4M Strain Generation by Reverse Genetics

The B/Bris PB1att 4M (E48K, K391E, E580G, and S660A) virus was generated by reverse genetics, as previously described [22], in co-cultured 293T and MDCK cells. Transfected cells were incubated at 35 °C for 24 h before the transfection media was replaced with Opti-MEM (Thermo Fisher Scientific, Waltham, MA, USA) containing 1 μg/mL tosylsulfonyl phenylalanyl chloromethyl ketone (TPCK)-trypsin (Worthington Biochemicals, Lakewood, NJ, USA) and 1% antibiotic/antimycotic solution (Sigma-Aldrich St. Louis, MO, USA). The B/Bris PB1 3M (K391E, E580G, S660A, and the HA tag) virus was rescued in our previous study [22]. Rescued viruses were amplified once in MDCK cells at 33 °C for 72 h (passage 1, P1). The B/Bris PB1att 4M virus for the animal study was grown from the P1 stock in SPF eggs for 72 h at 33 °C (egg passage 1, E1). Viral stocks were titrated by 50% tissue culture infectious dose (TCID_50_) or 50% egg infectious dose (EID_50_) using the Reed and Muench method [23], and sequenced by Sanger sequencing or next generation sequencing (NGS) to check for the presence of the inserted mutations and ensure integrity of the strain.

### 2.4. Sequencing

Sanger sequencing was performed commercially (Psomagen, Rockville, MD, USA), following the company’s recommendations, between the 5′-UTR (untranslated region) and the portion of the PB1 segment (up to approximately 2000 bp), which includes the four mutations. For Sanger sequencing, viral RNA was extracted from cell supernatants or allantoic fluids via the QIAamp Viral RNA Mini Kit (Qiagen, Germantown, MD, USA) and the PB1 segment was amplified in overlapping fragments by RT-PCR using Super Script III reverse transcriptase (Thermo Fisher Scientific, Waltham, MA, USA) using specific primers: 5′ AGCAGAAGCGGAGCCTTTAAG 3′ (B/Bris/PB1_UTR-F), 5′ AAGAGCGATTGCCACTGCTG 3′ (B/Bris/PB1_780_F), 5′ AAGGATGAAGAAACATGTATGGAAG 3′ (B/Bris/PB1_1448-F), and 5′ ATATCGTCTCGTATTAGTAGAAACACGAGCCTT 3′ (Bm-PB1b-2369R). PCR conditions were as follows: 55 °C for 2 min, 94 °C for 2 min, followed by 35 cycles at 94 °C for 30 s, 50 °C for 30 s, 68 °C for 3 min, and final elongation for 4 min at 68 °C.

For NGS, virus RNA samples were extracted from tissue culture supernatants and allantoic fluid via the MagNA Pure LC RNA Isolation Kit (Roche, Indianapolis, IN, USA) and multi-segment, one-step RT-PCR (MS-RT-PCR) was conducted for the amplification of the whole IBV genome, as previously described in [22], with the Superscript III High-Fidelity RT-PCR Kit (Thermo Fisher Scientific, Waltham, MA, USA). Libraries were prepared using the Nextera XT DNA Library Preparation Kit (Illumina, Inc., San Diego, CA, USA) according to the manufacturer’s instructions. The reaction was set up using 40% of the suggested final volume. Libraries were purified and size-selected using 0.7× Agencourt AMPure XP Magnetic Beads (Beckman Coulter Life Sciences, Indianapolis, IN, USA), and samples were normalized to 4 nM and pooled after fragment size distribution was analyzed using the High Sensitivity DNA Kit (Agilent, Santa Clara, CA, USA). Pooled libraries were loaded at 15 pM and sequenced using the 300-cycle MiSeq Reagent Kit v2 (Illumina, San Diego, CA, USA) in a paired-end 150-nucleotide run format (150 × 2). Full genome assembly was performed using a pipeline from previously published methods [24]. Briefly, low-quality sequences and adapters were removed by Cutadapt [25]. Quality-trimmed reads were mapped against reference sequences in the NCBI Influenza Research Database using STAR to filter chimeric reads. De novo assembly was performed using the Inchworm component of Trinity [26]. Final consensus sequences were generated using the Burrows–Wheeler alignment tool (BWA) [27] to map reads to the contigs and custom Perl scripts were used to confirm the most prevalent nucleotide at each position.

### 2.5. Genetic Stability of Viruses

B/Bris PB1 3M or B/Bris PB1att 4M viruses (MDCK P1) were serially passaged in MDCK cells and SPF eggs at 33 °C. Serial passages were performed until stability of the B/Bris PB1att 4M was confirmed, compared to the B/Bris PB1 3M virus, resulting in four serial passages in both MDCK (sP1–sP4) and in eggs (sE1–sE4). During each passage, viruses were inoculated at three different dilutions (10^−1^, 10^−2^, 10^−3^). Cell culture supernatants and allantoic fluids were harvested at 72 h post-inoculation (hpi). The highest dilution used for inoculation that showed the highest hemagglutination (HA) titers after 72 hpi was used for the subsequent passages. During each passage, virus from cell culture supernatants and allantoic fluids were subjected to Sanger sequencing and/or NGS as described above.

### 2.6. Viral Growth Kinetics In Vitro

MDCK cells at approximately 90% confluency in 24-well plates were infected at a multiplicity of infection (MOI) of 0.01 of each virus in Opti-MEM (Thermo Fisher Scientific, Waltham, MA, USA) containing 1 μg/mL TPCK-trypsin (Worthington Biochemicals, Lakewood, NJ, USA) and 1% antibiotic/antimycotic solution (Sigma-Aldrich St. Louis, MO, USA). Infected cells were incubated at 33 °C, 35 °C, and 37 °C under a 5% CO_2_ environment. Supernatants were collected at 6, 12, 24, 48, and 72 h post-inoculation (hpi). For titration of the samples, 50% tissue culture infectious dose (TCID_50_) titers were determined using the Reed and Muench method [23]. Viral growth kinetics were performed using B/Bris wild type (wt) strain as a control. The growth kinetics experiments were performed in triplicates.

### 2.7. Structural Analysis of the PB1 Segment

Three-dimensional (3D) analysis was conducted on the PB1 protein of the PB1 3M and PB1att 4M predicted sequences. DNAStar (Madison, WI, USA) was used to generate fasta files for the PB1 nucleotide sequences of B/Bris PB1 3M and B/Bris PB1att 4M; open reading frames (ORF) were identified with ExPASy (SIB bioinformatics resources) to produce the protein sequences. I-TASSER (University of Michigan, Ann Arbor, MI, USA) [28] and CHIMERA 1.141 (University of California, San Francisco, CA, USA) [29] were used to recreate the PB1 protein 3D structure and for analysis of amino acid distances.

### 2.8. Animal Use Compliance

The animal study was performed under animal biosafety level 2 (ABSL-2) containment and according to protocols approved by the University of Georgia’s Institutional Animal Use and Care Committee (IACUC). Mice had access to water and feed ad libitum. Mice that experienced significant weight loss (≥25%) or scored 3 or higher on a 4-point scale of disease severity were humanely euthanized according to IACUC-approved protocols and AVMA guidelines.

### 2.9. Vaccination and Vaccine Safety Study Design

The safety of B/Bris PB1att 4M was evaluated in DBA/2J mice (Charles River, Norwich, CT, USA) similarly to as previously described in [22]. Vaccination was performed using a prime-boost vaccination strategy, 3 weeks apart. Vaccinations were carried out via the intranasal (I.N.) route after mice were anesthetized with isoflurane. Mice (7 weeks-old, n = 36) were randomly divided into 3 groups (n = 12/group, 1/2 females). The B/Bris PB1att 4M group received a 50 μL inoculum containing 10^6^ EID_50_/mouse of B/Bris PB1att 4M, and was boosted by the same route with the same dose at 21 days post-vaccination (dpv). The two other groups were mock vaccinated with 50 µL 1× phosphate buffered saline (PBS, pH 7.4). After prime and boost, mice were monitored daily for clinical signs and survival up until the day prior to virus challenge. Body weight was monitored for up to 12 days post-vaccination and 12 days post-boost. On day 20 post-boost (41 dpv), 4 mice/group (n = 2 male, n = 2 female) were bled for antibody titer determination.

### 2.10. Virus Challenge Study Design

At 21 days post-boost (dpb), the mice in the B/Bris PB1att 4M vaccine group and the mice in one of the mock-vaccinated groups (mock-challenged) were challenged I.N. (n = 8 mice/group) with a 50 µL inoculum containing 10^7^ EID_50_/mouse of the lethal strain B/Bris PB2-F406Y [22]. The remaining mock-vaccinated group served as the negative control and mice were mock challenged with 50 µL of 1× PBS (mock-mock). After challenge, mice were monitored daily for clinical signs, body weight changes, and survival until 14 days post-challenge (dpc), at which time all remaining surviving mice were terminally bled via the facial vein under isoflurane anesthesia and humanely euthanized according to approved IACUC protocols and following AVMA guidelines.

### 2.11. Hemagglutination Inhibition Assay

Sera were treated with receptor-destroying enzyme (RDE) as follows: 15 μL of serum were mixed with 30 μL of 1× PBS and 15 μL of RDE and incubated at 37 °C overnight. The next morning, RDE was inactivated at 56 °C for 30 min. The treated sera were then brought to a final dilution of 1:10 with 1× PBS. The HI assay was performed as described previously in [30] using B/Brisbane/60/2008 as antigen and a 0.5% suspension of turkey red blood cells.

### 2.12. Statistical Analysis

Statistical analyzes were performed using GraphPad Prism software version 9 (GraphPad Software Inc., San Diego, CA, USA) using analysis of variance (ANOVA). A *p*-value below 0.05 was considered significant.

## 3. Results

### 3.1. Mutations E48K and K391E Remain Stable in Combination after Serial Passages in MDCKs and SPF Eggs

The B/Bris PB1 3M and B/Bris PB1att 4M strains were serially passaged at 33 °C in MDCK cells (sP1–sP4) and SPF eggs (sE1–sE4; Table 1). Sanger sequencing analysis of the PB1 segment confirmed that the B/Bris PB1att 4M virus stably retained all modifications introduced (E48K, K391E, E580G, and S660A) after four serial passages in MDCK cells and in SPF eggs. In contrast, two new mutations were identified in the PB1 of the B/Bris PB1 3M virus at passage sE4 (E48K and G161C), but not at sP1 or sP4 (MDCK cell passages).

### 3.2. Stability of B/Bris PB1att 4M Strain Was Confirmed after Serial Passages

High-throughput sequencing of the viral genome of B/Bris PB1att 4M further confirmed that key mutations (E48K, K391E, E580G, and S660A) were maintained after four passages in both MDCK cells and SPF eggs. As previously described for a similar IBV LAIV backbone [22], significant genomic stability was observed in the B/Bris PB1att 4M strain (Table 2).

### 3.3. B/Bris PB1att 4M Virus Shows a Temperature-Sensitive Phenotype In Vitro

**Figure 1:**
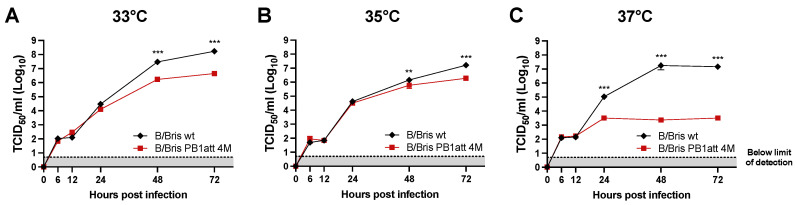
B/Bris PB1att 4M displays a temperature-sensitive phenotype in growth kinetics in vitro. MDCKs cells were inoculated with an MOI of 0.01 with B/Bris PB1att 4M and B/Bris wildtype (wt) strains and incubated at 33 °C (**A**), 35 °C (**B**), and 37 °C (**C**). Supernatants were collected at 6, 12, 24, 48, and 72 h post-infection and viral titers were quantified by TCID_50_ assays. The growth kinetics experiments were performed in triplicate and results represent an average of all experiments. Two-way ANOVA corrected for multiple comparisons using Sidak’s test was used to calculate *p* values. ** *p* < 0.01, *** *p* < 0.001. Plotted data represent means ± standard errors.

To evaluate the temperature-sensitive (ts) phenotype of the B/Bris PB1att 4M strain, growth kinetics were evaluated in confluent MDCK cells at 33 °C, 35 °C, and 37 °C and compared with the B/Bris wt virus. The B/Bris PB1att 4M and B/Bris wt viruses grew to similar titers at 33 °C and 35 °C (Figure 1). The B/Bris wt strain displayed higher titers compared with the B/Bris PB1att 4M strain at 48 hpi and at 72 hpi at all temperatures, but titers were much higher at 37 °C. Overall, the B/Bris PB1att 4M strain presented a ts phenotype (Figure 1).

### 3.4. Structural Analysis of PB1 from B/Bris PB1att 4M Suggests Molecular Interactions between Amino Acid Residues at Positions 48 and 391

**Figure 2:**
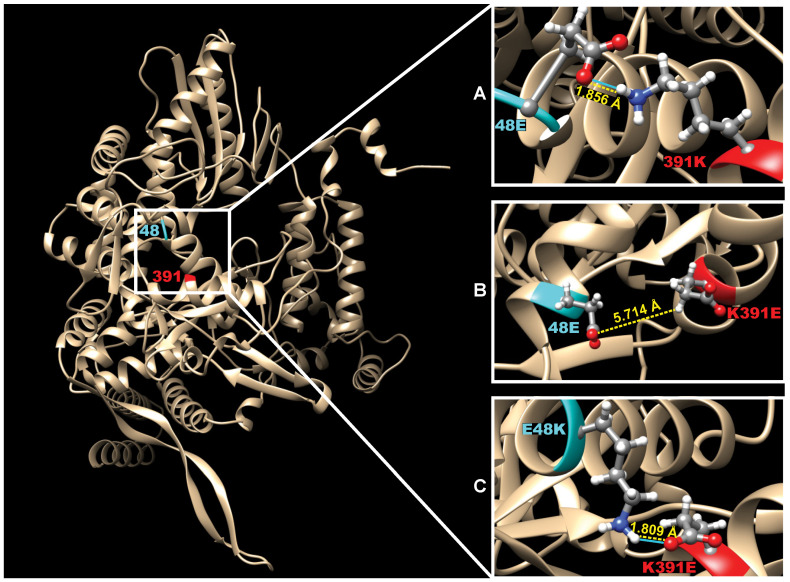
Predicted 3D structure of PB1 showing mutations in residues 48 and 391 and respective molecular interactions between them. Three-dimensional structure predictions using iTASSER of the B/Bris PB1 protein sequence and potential interactions at the interface of amino acids 48 and 391. Blue lines represent the presence of hydrogen bonds. Dotted yellow lines indicate predicted distances in angstroms (Å). (**A**) Potential interaction via a single hydrogen bond and a predicted distance of 1.856 Å between amino acids 48E and 391K in the wt PB1. (**B**) Lack of predicted hydrogen bonds and a predicted distance of 5.714 Å between amino acids 48E and 391E in the PB1 3M protein. (**C**) Amino acids 48K and 391E restore the predicted single hydrogen bond interaction and a distance of 1.809 Å between these two amino acids in the PB1att 4M protein.

To visualize molecular interactions between the individual mutations/residues, the predicted 3D structure of the PB1 protein was recreated (Figure 2). The amino acid in position 48 is located on one of the alpha helixes facing the amino acid in the 391 position. The wild type B/Bris PB1 protein structure showed potential interaction between the amino acids 48E and 391K via a single hydrogen bond or salt bridge, with a predicted distance between the two residues of 1.856 Å. In contrast, in the PB1 3M predicted structure, no hydrogen bonds or any notable interactions between the side chain of the wild type amino acid 48E and the 391E mutation were noted. In the PB1 3M protein, the length between the most proximate hydrogen and oxygen groups capable of forming salt bridges was 5.714 Å. However, in the PB1att 4M, there was indication of potential interaction between 48K and 391E via a single hydrogen bond/salt bridge, and the 1.809 Å distance between the two resembles the distance between E48 and K391 in the wild type PB1, suggesting a stronger interaction and potential effect on protein stability.

### 3.5. B/Bris PB1att 4M Is Safe and Immunogenic as a Potential Vaccine Candidate

**Figure 3:**
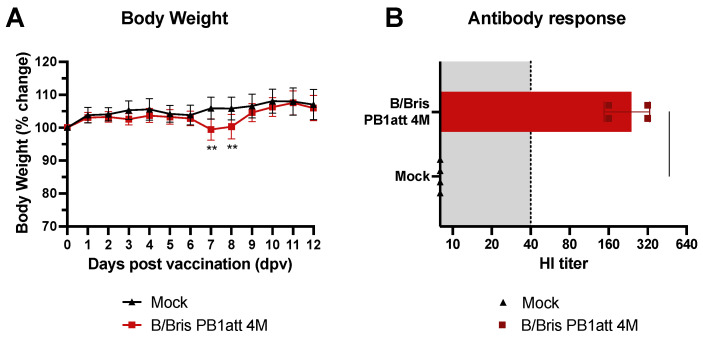
Safety and immunogenicity of the B/Bris PB1att 4M virus. DBA/2J 4-week-old, male and female mice were inoculated I.N. with either B/Bris PB1att 4M or PBS (mock group). (**A**) Percentage body weight change was evaluated daily for 12 days following vaccination. (**B**) Immunogenicity of the B/Bris PB1att 4M virus was measured by HI assay in mouse sera at 20 days post-boost (dpb). Shaded area indicates cutoff for HI titers considered to be predictive of protection. Weight loss data were compared using two-way ANOVA corrected for multiple comparisons using Holm–Sidak’s test. Significant differences, compared with mock group, are indicated. ** *p* < 0.01. Plotted data represent means ± standard errors.

To test the safety and immunogenicity of the B/Bris PB1att 4M vaccine strain in vivo, DBA/2J mice were vaccinated with either the B/Bris PB1att 4M virus (n = 12) or mock-vaccinated (1× PBS; n = 24) by intranasal inoculation in a prime-boost regimen, 21 days apart. Mice vaccinated with B/Bris PB1att 4M demonstrated minor weight loss starting at 7 dpv, but quickly recovered (Figure 3A). No body weight loss was observed after boost. No other clinical signs were observed, and all mice were apparently healthy during the post-vaccination period. HI titers were measured at 20 dpb and vaccinated mice showed titers ≥160, above the level of 40 that is considered predictive of protection against clinical disease (Figure 3B) [31].

### 3.6. B/Bris PB1att 4M Vaccine Candidate Successfully Protects Mice against Lethal Challenge

**Figure 4:**
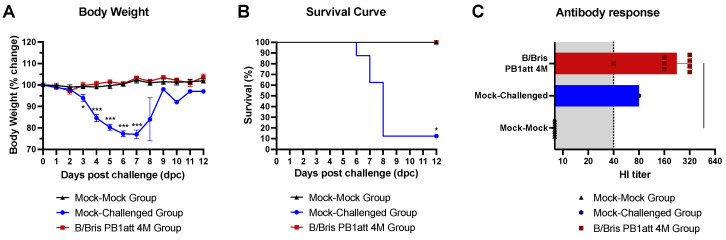
Protective efficacy of B/Bris PB1att 4M vaccine against homologous challenge. Vaccinated (B/Bris PB1att 4M) or unvaccinated (mock-challenged) DBA/2J mice were challenged with the homologous lethal strain B/Bris PB2-F406Y 21 days post-boost. A non-vaccinated, non-challenged control group was included (mock-mock). (**A**) Percentage body weight change and (**B**) survival rate were evaluated daily for 12 days post-challenge. (**C**) The serum antibody response against the homologous (B/Bris wt) virus was measured by HI assay at 14 days post-challenge (dpc). Shaded area indicates cutoff for HI titers considered to be predictive of protection. Weight loss data were compared using two-way ANOVA corrected for multiple comparisons using Holm–Sidak’s test. Survival curves were compared using log-rank Mantel–Cox curve comparison. Significant differences, compared with the mock-challenged group, are indicated. * *p* < 0.05, *** *p* < 0.001. Plotted data represent means ± standard errors.

In order to assess the efficacy of the B/Bris PB1att 4M vaccine strain, mice were challenged at 21 dpb with a lethal dose (10^7^ EID_50_/mouse) of the B/Bris PB2-F406Y mutant strain [22]. A group of mock-vaccinated mice remained unchallenged throughout the study (mock-mock). Mice in the B/Bris PB1att 4M-vaccinated group showed no apparent signs of disease with minimum weight loss, and all survived the virus challenge (Figure 4A,B). Mice from the mock-challenge group showed rapid and severe weight loss along with severe clinical signs and approximately 87% of the mice in this group succumbed or had to be humanely euthanized by 8 dpc (Figure 4A,B). There was one survivor out of eight, a female mouse that also showed severe weight loss but started to recover at 7 dpc and demonstrated rapid recovery starting at 8 dpc (Figure 4A,B). As expected, HI antibody titers against B/Bris PB1att 4M remained high in vaccinated mice at 14 dpc (Figure 4C). The survivor mouse from the mock-challenge group seroconverted as well (Figure 4C).

## 4. Discussion

LAIVs are generally thought to elicit superior immunity and cross-protection against influenza viruses compared with inactivated products due to the perceived notion that they can better stimulate cell-mediated and mucosal immune responses in addition to humoral systemic immunity [32,33]. However, in recent years, the overall low performance of LAIVs in some age groups highlights the need for the continued development of more efficacious alternative LAIV technologies. We have developed a LAIV strategy by incorporating ts mutations on the PB1 that can be applied similarly for both IAV and IBV [19,21,22]. This strategy was shown to be safe and effective in mice, pigs, and chickens, stimulating cross-protective immunity against antigenically distinct viruses, and can be used in a quadrivalent formulation [19,20,22,34,35,36]. In a previous study using an IBV LAIV candidate with the PB1 gene segment carrying three ts mutations—K391E, E580G, and S660A—and a C-terminus HA tag, the K391E mutation reverted to its wild type form (E391K) during the initial passages in SPF eggs [22]. In contrast, passage in MDCK cells led to the quick emergence of the E48K mutation and retention of the K391E. Compensatory mutations are thought to emerge when the initial fitness of the mutant strain is reduced [37]. Our initial observations suggested that the E48K mutation could stabilize the ts K391E mutation in the vaccine candidate strain and, therefore, could contribute to a more stable and safe attenuated vaccine candidate. In this report, we found that the K391E mutation was indeed maintained in the presence of the E48K mutation, and the resulting B/Bris PB1att 4M strain was attenuated and safe, resulting in efficacious protection against homologous challenge.

Genomic analysis of the B/Bris PB1att 4M strain after four passages in eggs revealed the stability of the E48K, K391E, E580G, and S660A mutations. In contrast, the B/Bris PB1 3M strain passaged four times in eggs showed the presence of not only the intended mutations (K391E, E580G, and S660A) but also the acquisition of the potentially compensatory mutation E48K and the additional G161C mutation whose role remains unknown. Next generation sequencing (NGS) revealed all the target mutations were maintained in the PB1 of B/Bris PB1 4M strains, and there were no amino acid mutations in other segments. This reveals that the viral genome of the B/Bris PB1 4M strain is stable even after several passages in MDCKs and SPF eggs, indicating the introduction of the compensatory mutation E48K does not significantly alter the overall genome structure.

Compensatory mutations may contribute to improved virus fitness by either inducing conformational changes towards either a more energetically favorable form of the protein and/or by altering molecular bonds [38,39]. Forming and/or increasing hydrogen bonds among key amino acid residues is a known effector of protein stability [40,41]. In addition, E and K amino acid residues can form salt bridges if the distance between their side chains is <4 Å. Three-dimensional structure prediction analysis of the IBV PB1 protein suggests that the 48E–391K pair are part of opposite alpha-helixes occupying a space that would favor formation of a single hydrogen bond and/or salt bridge (approximately 1.8 Å). This is not the case for the 48E–391E pair in the PB1 3M protein (distance >5 Å); however, the potential for a hydrogen bond/salt bridge is restored in the 48K–391E pair in the PB1att 4M protein (approximately 1.8 Å). The stability of the B/Bris PB1att 4M strain in either eggs or MDCK cells and the acquisition of the E48K mutation in the egg-passaged B/Bris PB1 3M is consistent with the notion of a stabilizing role of the E48K that maintained the K391E mutation.

Interestingly, a similar number of passages in MDCK cells did not affect the stability of the PB1 gene segment in the B/Bris PB1 3M strain, and the E48K mutation was not observed. This finding was unexpected considering prior observations with our original IBV LAIV candidate in which the E48K mutation was initially identified after the first serial passage in MDCK cells [22]. Another unexpected finding was the E48K mutation emerging and stabilizing the K391E mutation during serial passages in SPF eggs. A key difference between the previous results and those in this report is that the virus stocks used for serial passages here were amplified in MDCK cells, while in the previous study they were initially amplified in MDCK cells followed by amplification in eggs. Another difference is the absence of the HA tag at the C-terminus of the PB1 protein gene segment in the B/Bris PB1att 4M strains. It is important to note that in our previous work, a virus rescued without the HA tag also showed the PB1 E48K mutation that seemed to stabilize the K391E mutation after nine serial passages in eggs [22]. It is tempting to speculate that the C-terminal HA tag further impairs the polymerase function of the PB1 subunit protein forcing the K391E mutation to quickly revert to wild type when passaged in eggs. Regardless of the potential bottleneck effect of the HA tag for PB1 activity and the effect of initial virus stock amplification in eggs, our results strongly suggest a functional link between amino acids 48 and 391 and significant stability of the other two ts mutations (E580G, S660A) in the PB1 protein of IBV [22]. The E48K mutation was only found in 3 natural isolates as revealed by the analysis of 1699 unique complete IBV PB1 protein sequences deposited in the Influenza Research Database (IRD; www.fludb.org (accessed on 2 July 2021)). Furthermore, the B/Bris PB1att 4M strain demonstrated remarkable genomic stability in the rest of the genome (Table 2), a finding that is consistent with our previous study [22].

In vitro kinetics studies confirmed that the B/Bris PB1att 4M strain retained its ts phenotype despite the E48K mutation. In vivo, in DBA/2J mice, the B/Bris PB1att 4M was attenuated and safe and provided protection against homologous challenge. DBA/2J mice primed with a high dose of the B/Bris PB1att 4M strain showed minor weight loss between 3–8 days post-vaccination and quickly recovered, confirming its attenuation and safety in vivo. Female and male mice were equally protected against a lethal dose of the antigenically homologous B/Bris/PB2 F406Y strain, exhibiting no weight loss and no mortality. In contrast, the non-vaccinated B/Bris/PB2 F406Y-challenged mice showed significant signs of disease, weight loss, and mortality, and only one non-vaccinated mice survived the virus challenge.

## 5. Conclusions

We provide evidence of the molecular basis for a compensatory mutation in the PB1 protein of an IBV LAIV candidate that improves its stability and likely reduces the risk for reversion to virulence, while retaining the safety and efficacy profiles expected in an attenuated influenza vaccine candidate.

## Figures and Tables

**Table 1:** Evaluation of genetic stability in the PB1 gene of B/Bris PB1 3M and B/Bris PB1att 4M strains after serial passage in MDCK cells and SPF eggs. Newly acquired mutations during the serial passages that were not introduced via reverse genetics are highlighted in bold. All mutations were confirmed by Sanger sequencing. sE1 and sE4 represent the 1st and 4th serial passage in SPF eggs, respectively. sP1 and sP4 represent the 1st and 4th passage in MDCK cells, respectively.

**Strain**	**SPF Egg Passage**	
	**sE1**	**sE4**
B/Bris PB1 3M	K391E, E580G, S660A	**E48K**, K391E, E580G, S660A, **G161C**
B/Bris PB1att 4M	E48K, K391E, E580G, S660A	E48K, K391E, E580G, S660A
**Strain**	**MDCK Passage**	
	**sP1**	**sP4**
B/Bris PB1 3M	K391E, E580G, S660A	K391E, E580G, S660A
B/Bris PB1att 4M	E48K, K391E, E580G, S660A	E48K, K391E, E580G, S660A

**Table 2:** Amino acid substitutions in all gene segments of B/Bris PB1att 4M after serial passage. Predicted mutations indicate mutations introduced into the PB1 segment via site-directed mutagenesis and reverse genetics.

Segments	Predicted Mutations	Viral Stock Amplified in MDCK Cells (P1)	Serially Passaged in MDCKS (sP4)	Serially Passaged in SPF Eggs (sE4)
PB2	None	None	None	None
PB1	E48K (g163a) K391E (a1192g) E580G (a1760g) S660A (t1999g)	E48K (g163a) K391E (a1192g) E580G (a1760g) S660A (t1999g) Stop ^a,s^ (g2280a)	E48K (g163a) K391E (a1192g) E580G (a1760g) S660A (t1999g) Stop ^a,s^ (g2280a)	E48K (g163a) K391E (a1192g) E580G (a1760g) S660A (t1999g) Stop ^a,^^s^ (g2280a)
PA	None	None	None	None
HA	None	None	None	None
NP	None	None	None	None
NA	None	None	None	None
NB	None	None	None	None
BM1	None	None	None	None
BM2	None	None	None	None
NS1	None	None	None	None
NS2/NEP	None	None	None	None

^a^ mutation identified at the stop codon of the open reading frame that was present in the viral stock; ^s^ synonymous mutation; higher case letters, amino acids; lower case letters, nucleotides.

## Data Availability

The data presented in this study are available within the article.
